# The Uptake of Ivermectin and Its Effects in Roots, Leaves and Seeds of Soybean (*Glycine max*)

**DOI:** 10.3390/molecules25163655

**Published:** 2020-08-11

**Authors:** Martina Navrátilová, Lucie Raisová Stuchlíková, Kateřina Moťková, Barbora Szotáková, Lenka Skálová, Lenka Langhansová, Radka Podlipná

**Affiliations:** 1 Department of Biochemical Sciences, Faculty of Pharmacy in Hradec Králové, Charles University, Heyrovského 1203, 500 05 Hradec Králové, Czech Republic; navratimart@faf.cuni.cz (M.N.); stuchli2@faf.cuni.cz (L.R.S.); szotakova@faf.cuni.cz (B.S.); skaloval@faf.cuni.cz (L.S.); 2 Laboratory of Plant Biotechnologies, Institute of Experimental Botany, Czech Academy of Sciences, 165 02 Praha 6-Lysolaje, Czech Republic; motkova@ueb.cas.cz (K.M.); langhansova@ueb.cas.cz (L.L.)

**Keywords:** anthelmintics, biotransformation, drug metabolites, antioxidant enzymes, isoflavonoids

## Abstract

In recent years interest has grown in the occurrence and the effects of pharmaceuticals in the environment. The aim of this work is to evaluate the risk of fertilizing crops with manure from livestock treated with anthelmintics. The present study was designed to follow the fate of the commonly used anthelmintic drug, ivermectin (IVM) and its metabolites in soybeans (*Glycine max* (L.) Merr.), a plant that is grown and consumed world-wide for its high content of nutritional and health-beneficial substances. In vitro plantlets and soybean plants, cultivated in a greenhouse, were used for this purpose. Our results showed the uptake of IVM and its translocation to the leaves, but not in the pods and the beans. Four IVM metabolites were detected in the roots, and one in the leaves. IVM exposure decreased slightly the number and weight of the beans and induced changes in the activities of antioxidant enzymes. In addition, the presence of IVM affected the proportion of individual isoflavones and reduced the content of isoflavones aglycones, which might decrease the therapeutic value of soybeans. Fertilization of soybean fields with manure from IVM-treated animals appears to be safe for humans, due to the absence of IVM in beans, the food part of plants. On the other hand, it could negatively affect soybean plants and herbivorous invertebrates.

## 1. Introduction

Nowadays, veterinary drugs represent serious contaminants of the environment [1]. Drugs, regularly used in livestock, enter the environment directly via excrements on the pastures, or via manure from treated animals. The use of manure, recommended for sustainable farming [2], has many advantages in terms of mineral or nitrogen sequestration, but it does bring a risk of spreading veterinary drugs into farmland. As a result of the poor metabolism of drugs applied in farm animals, more than 50% of these drugs eventually reach soil or water [3]. Another way veterinary drugs are introduced into the environment is through the use of biosolids, produced in the utilization of wastewater as fertilizer on arable land [4]. The uptake and translocation of pharmaceuticals from soil and water into plant tissues has been documented in several studies [5]. Plants have the ability to metabolize pharmaceuticals and/or accumulate them or their metabolites in different tissues, including fruits [6,7]. This accumulation would potentially pose a clear risk to human health. The quantitative model proposed by [8] offers a comprehensive overview of pharmaceutical contamination through the food production system.

Antiparasitic drugs are widely used to both prevent and treat animal parasitic diseases in livestock. The macrocyclic lactone ivermectin (22,23-dihydroavermectin B_1a_ and 22,23-dihydroavermectin B_1b_; IVM) is a very effective drug against endo/ectoparasites, in both animals and humans [1]. In the organism, this compound is poorly metabolized and largely secreted in the bile and excreted into the feces [9]. Once in dung, IVM, as well as other drugs, can leach into soil and affect non-target (soil) invertebrates, or other organisms. The concentration of IVM in feces, after a single subcutaneous dose of 0.2 mg·kg^−1^ b.w. was determined to be 0.87 μg·g^−1^ dry weight for cattle, and 0.93 μg·g^−1^ for sheep [10]. The uptake of IVM from soil by plants growing near contaminated feces has been described in [9]. Our previous experiments on *Sinapis alba* revealed significant phytotoxicity of IVM [10]. When the uptake and metabolism of IVM was studied in the model plant *Arabidopsis thaliana*, the parent compound was found in both the roots and rosettes, with six metabolites detected in the roots. IVM exposure caused changes in the transcription level of numerous genes involved in the response to salt, osmotic and water deprivation stress, in ion homeostasis, as well as in defense responses to pathogens [11].

In addition to the negative effect on plant physiology, the presence of low concentrations of anti-parasitic agents in fodder plants may also encourage the development of resistant strains of parasites in livestock [12,13]. These possibilities are similar and relevant to the potential development of drug resistance in helminths that affect humans [14,15]. However, the study of the presence and effects of anti-parasitic drugs and their metabolites in crop plants has been largely neglected.

For these reasons, we decided to study the uptake, distribution and metabolism of IVM in soybean plants. The soybean (*Glycine max*) was selected due to its widespread cultivation around the world and its nutrient importance. We sought to determine if IVM from the manure of treated animals can make its way to beans consumed by humans, a trajectory which could potentiate the development of the resistance of human parasites. Besides its nutritional value, various health-benefits have been ascribed to the soybean, particularly its isoflavones daidzein, glycitein, and genistein. In fact, 99% of the isoflavonoid compounds in soybeans are found in their glucosides (daidzin, glycitin, genistin). The bioavailability of the active compounds after consumption depends on many factors, one of which is the composition of the intestinal microbiota [16]. After the consumption of soybeans, the glucosides are converted by digestive or microbial enzymes to their corresponding isoflavones with great biological effects, e.g., anti-atherosclerotic, estrogenic and anticancer properties. Because anthelmintics are metabolized in plants mainly by oxidation, reduction and glycosylation, we hypothesize that anthelmintics may affect the level of flavonoids, due to competition for enzymes that are used in their synthesis. Therefore, the effects of IVM on the content of individual isoflavones in soybeans were also studied. In addition, the effects of IVM on soybean growth and yield were monitored, and antioxidant enzyme activities were tested.

## 2. Results and Discussion

In our previous study, we demonstrated the uptake of IVM by *Arabidopsis thaliana* and the effect on its transcriptome [11]. Based on the results obtained in this model plant, we studied the uptake and accumulation of IVM in soybeans (*Glycine max*, a common crop abundantly fertilized with manure). We sought to determine if the IVM present in manure from treated animals could be eventually translocated in beans consumed by humans, which could potentiate the development of resistance in human parasites. In addition, we monitored the effect of IVM on plant growth, fitness, yield, antioxidant enzymes activities as well as isoflavones content to obtain more complex information about IVM impacts in crops.

### 2.1. Effect of IVM on Soybean Growth and Yield

Our results showed that the growth of soybean plants was not visibly affected by the application of IVM, but the yield of beans was lower, i.e., the number of seeds and their average weight was lower in the IVM treated plants (7.6 ± 0.8 seeds weighed 1.21 ± 0.09 g) than in the controls (10.5 ± 1.6 seeds weighed 1.79 ± 0.3 g). These results indicate that IVM (e.g., in manure or biosolids) may have an adverse effect on soybean yield.

### 2.2. Uptake and Metabolism of IVM in Soybeans

**Figure 1:**
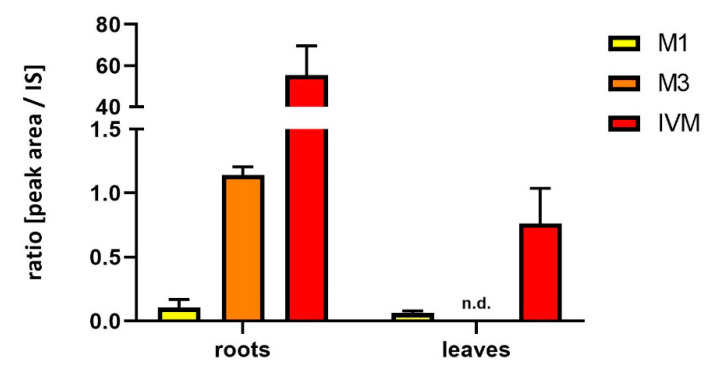
Relative amount of IVM and its main metabolites (M1, M3) in the roots and leaves of soybean plants after 42 days of treatment. Data are expressed as mean ± SD (*n* = 6), n.d. = not detected.

**Figure 2:**
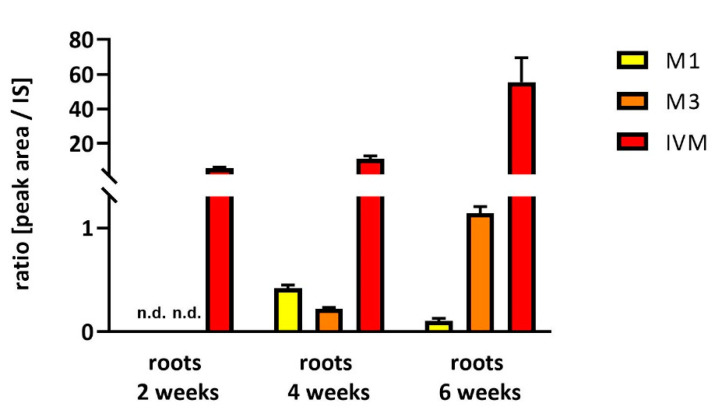
Time dependence of IVM uptake and metabolites formation in soybean roots. Relative amount of IVM main metabolites (M1 and M3) and IVM. Data are expressed as mean ± SD (*n* = 6), n.d. = not detected.

Soybeans cultivated in a greenhouse and watered with 10 µM IVM solution for 42 days were able to uptake IVM, and transport it to the leaves, but not to the pods and seeds. The parent drug IVM was detected at *m*/*z* 892 [M + NH_4_]^+^ (t_R_ = 13.9 min). Four metabolites of IVM formed via hydroxylation (+O) and demethylation (-CH_2_) were found. The detected metabolites were identified based on the presence of protonated molecules with ammonium adducts [M + NH_4_]^+^ in positive ion mode. Four metabolites were found in the roots (M1–M4), and only one (M1) in the leaves. The molecular weight, retention time and fragmentation ions of all the identified metabolites are shown in Table 1. The distribution of IVM, and its main metabolites in individual parts of soybeans, is presented in Figure 1. The time-dependent uptake of IVM and the formation of metabolites in roots is demonstrated in Figure 2.

Comparing the metabolism of IVM in *G.max* and *Arabidopsis thaliana*, more metabolites (6) were formed in *A. thaliana* [11], but they were present only in the roots, while one IVM metabolite was found also in the leaves. However, no IVM conjugates were found in *G. max* or in *A. thaliana*.

According to the chemical structure, IVM metabolites found in *G. max* appear to be relatively stable substances. Thus, the decomposition back into parent compound in the gastrointestinal tract of consumers (invertebrates, herbivores) is unlikely. Nevertheless, the amount of metabolized IVM is very low, and unmetabolized IVM predominated in all soybean parts, and may negatively affect consumers of soybean roots and leaves. Still, our most important finding is that IVM and its metabolites do not occur in soybean seeds, i.e., in the human food-relevant part of the plant. Therefore, this anthelmintic drug cannot find a direct way into human food.

IVM is used for the treatment of various human parasitosis, including head lice, scabies, river blindness (onchocerciasis), strongyloidiasis, trichuriasis, ascariasis, and lymphatic filariasis. In view of the absence of IVM and its metabolites in the beans, the use of manure with IVM-traces for soybean fields fertilization and subsequent beans consumption by infected people does not promote drug-resistance development in human parasites.

### 2.3. The Effect of IVM on Antioxidant Enzyme Activities in Soybeans

**Figure 3:**
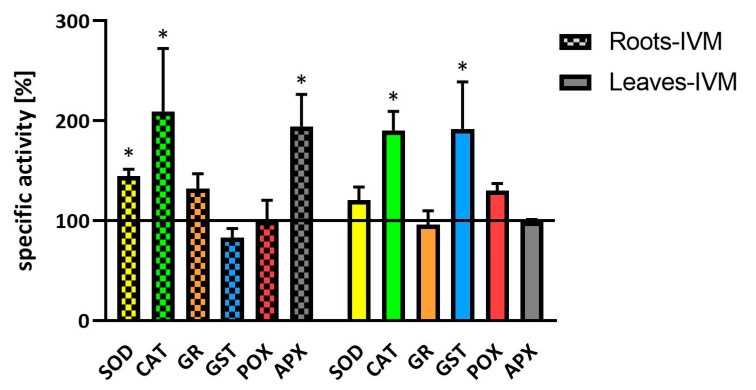
Specific activities of antioxidant enzymes (SOD, CAT, GR, GST, POX, and APX) in soybean roots and leaves after seven days of exposure to IVM. Data are presented as the percentage of specific enzyme activity of control plants (100%). Data are expressed as mean ± SD (*n* = 4). * indicates statistically significant difference between treated and control plants.

The exposure of a plant to pharmaceuticals could lead to oxidative stress, e.g., the increased production of total reactive oxygen species (ROS) as described in *Nicotiana tabacum* cells exposed to diclofenac [17]. Plants possess various defense mechanisms to cope with oxidative stress, one of which is an increase in the activities of antioxidant enzymes. In the present study, therefore, we compare the activity of several antioxidant enzymes, superoxide dismutase (SOD), catalase (CAT), glutathione reductase (GR), glutathione S-transferase (GST), guaiacol-specific peroxidase (POX), and ascorbate peroxidase (APX), in plants cultivated in vitro in a medium supplemented by IVM (10 µM) and in the control plants (Figure 3).

Like other authors [17,18], we used in vitro plants to avoid interference with the side effects of microbial presence. After the exposure of plants to ivermectin, the activity of CAT was significantly increased in both the roots and leaves; the activity of APX was significantly increased in the roots, and SOD activity was slightly enhanced in the roots. Similarly, [19] has described the increased activity of these antioxidant enzymes in duckweed fronds affected by amoxicillin. In addition, other antibiotics enhanced the activity of SOD and APX in wheat [20]. GST, the main enzymes of second phase of biotransformation of xenobiotics in plants, showed increased activity only in leaves. The increase of GST activity in the plantain was also observed under fenbendazole treatment [21].

### 2.4. The Effect of IVM on Isoflavones Content in Soybeans

Our results showed that IVM was absorbed by soybean roots and transported to the leaves. During this time, IVM was partly metabolized and both the parent IVM and its metabolites could affect the plant’s metabolism of endogenous compounds. As the synthesis of plant polyphenols react extremely sensitively to the presence of various stress factors (including pharmaceuticals), we hypothesized that IVM could change the amount of polyphenols accumulated in seeds. During our experiment, the total amount of polyphenols in the seeds from the IVM-exposed and the control plants were assayed spectrophotometrically. At the end of the experiment, we harvested the mature seeds and measured the concentration of individual polyphenols using HPLC.

**Figure 4:**
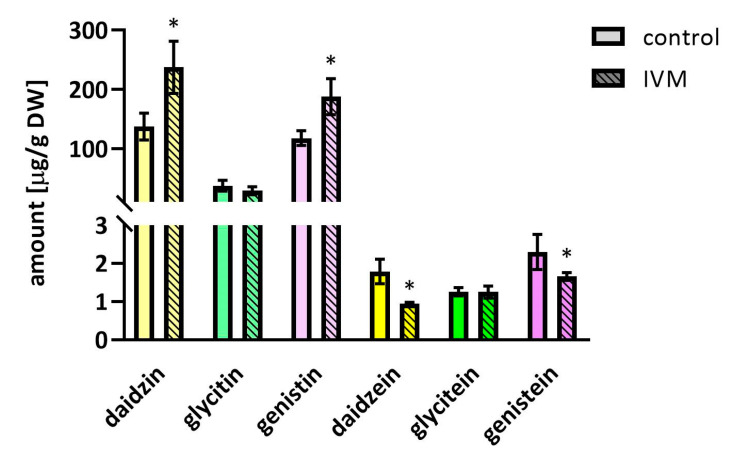
Concentration of isoflavones in dry seeds harvested after 42 days of the experiment. The final concentration of IVM added to the soil was 35 µg/g of dry soil. Data are expressed as mean ± SD (*n* = 6). * indicates statistically significant difference between treated and control plants.

As isoflavonoids play a vital role in plant defense response against various biotic and abiotic stresses [22], it was not surprising that the total amount of polyphenols increased in the IVM stressed plants (444.6 µg/g vs. 298.4 µg/g in control plants). In addition, as described in [23], the changes in the amount and the composition of isoflavones in soybeans depend on the temperature during the vegetation period. Along with the increase of total polyphenol content, we observed significant IVM-induced changes in the ratio of isoflavones and their glycosylated forms, with the results (presented in Figure 4) showing that IVM increased the content of glycosides daidzin and genistin, but decreased the content of the aglycones daidzein and genistein. One of the possible causes might be the IVM-induced increased activity of glycosyltransferases. We have previously described the up-regulation of the expression of seven glycosyltransferases in *Arabidopsis thaliana* under IVM exposure [11,24].

**Figure 5:**
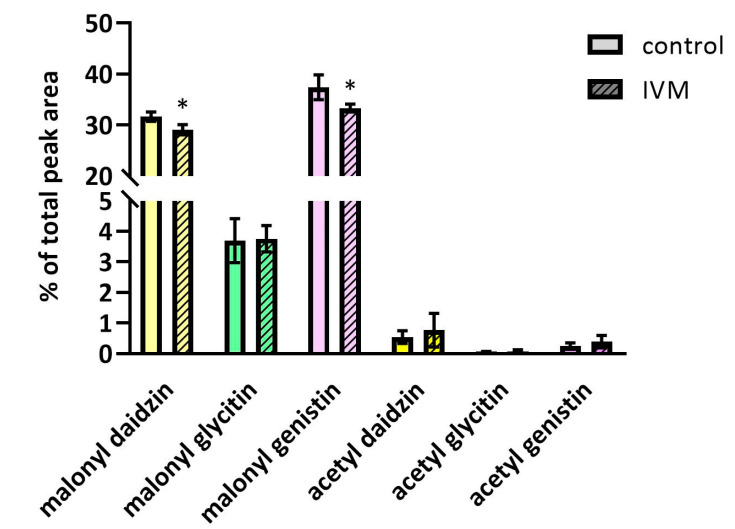
The quantity changes of malonylated and acetylated forms of daidzin, glycitin and genistin under IVM exposition. Data are expressed as mean ± SD (*n* = 6). * indicates statistically significant difference between treated and control plants.Although IVM has been shown to increase the total polyphenol content in soybean seeds, it reduced the content of isoflavones aglycones, which are considered as more effective in preventing certain hormone-dependent and other diseases [26]. Thus, the presence of IVM in manure, and subsequently in soybeans, may decrease the therapeutic value of soybean.

In soybeans, IVM exposure also led to a slight decrease in malonyl-daidzin and malonyl-genistin (see Figure 5), indicating that IVM might also affect the expression and activity of malonyltransferases. The acetylated forms of daidzin, glycitin and genistin were also identified, although their concentrations were only slightly above the detection limit, and changes between the exposed and control plants were not observed. The content of glycitin and glycitein as well as malonyl-glycitin was the same in the treated and control plants. The enhanced production of glycosidic isoflavones (daidzin, malonyl-daidzin, and malonyl-genistin) under methyl jasmonate treatment (a signal molecule) has been described previously [25].

## 3. Materials and Methods

### 3.1. Cultivation of Soybean Plants in a Greenhouse

Seeds of soybean (*Glycine max* (L.) Merr. cv, Moravia) were obtained from AGRITEC Research, Breeding and Services, Ltd. (Šumperk, Czech Republic). The germinated seeds were planted into soil supplemented by nitrazon (*Rhizobium* mix; Farma Žiro, s.r.o., Nehvizdy, Czech Republic) and grown in a greenhouse for one month (23 °C, with relative humidity of about 60%). The plants were irradiated using sodium discharge lamps (400 W, ZG Lighting, Prague, Czech Republic s.r.o.) with a 12 h photoperiod and an average irradiance of 72 μmol·m^−2^·s^−1^ on the plant surfaces, with the horizontal differences in irradiance < 20%. For the experiments only well-grown plants were chosen.

In the first experiment, the time dependence (2, 4, and 6 weeks) of IVM uptake was monitored. Six pots with soybean plants were irrigated 3-times a week with a 10 µM solution of IVM (pre-dissolved in DMSO) in nutrient medium. After 2 and 4 weeks, only the roots and leaves were harvested, as the plants at this time have no pods and seeds yet. After 6 weeks the roots, leaves and pods with seeds were harvested, and the ratio of IVM content and the contents of its metabolites were determined. After 6 weeks, the final amount of IVM added in the soil was 35 µg IVM/g dry soil.

In the second experiment, 6 soybean plants were irrigated 3 times a week with a 10 µM solution of IVM (pre-dissolved in DMSO) in nutrient medium for 6 weeks. The 6 control plants were watered with nutrient solution supplemented by DMSO in the same concentration as the experimental plants. At the end, only the mature seeds were collected, and the content of polyphenols was measured both in the IVM treated and control plants.

### 3.2. In Vitro Cultivation of Soybean Plants

The in vitro plantlets from the stock culture were transferred on a solid B5 medium [27] and grown under defined conditions (temperature 25 °C, 16-h photoperiod, irradiance of 72 μmol·m^−2^·s^−1^) for 7 days. Then the plantlets were replanted on media supplemented with IVM (pre-dissolved in DMSO) to achieve 10 µM concentration. The control medium was treated with pure DMSO, in corresponding concentrations. The plants were harvested after 7 days, with the roots and leaves of plants collected separately and stored in a freezer (−80 °C). All the experiments were performed in triplicate.

### 3.3. HPLC Analysis of Isoflavones

After six weeks of incubation, the seeds from the control and IVM treated plants were harvested for an analysis of isoflavones (daidzin, daidzein, genistin, genistein, glycitin, and glycitein). Our method of sample preparation and isoflavone content determination was based on literature [28,29,30]. The seeds were lyophilized and ground in a laboratory mill, then homogenized in liquid nitrogen and extracted with chilled 80% methanol (100 mg/mL) for 24 h by shaking and in an ultrasonic bath (3 × 30 min). The extracts were centrifuged (4000 g, for 15 min at 24 °C) and filtered (0.2 µm) before the HPLC analyses.

The content of daidzin, daidzein, genistin, genistein, glycitin, and glycitein was determined by HPLC (Alliance HPLC System, Waters, Milford, MA, USA), equipped with a reverse phase SiC_18_Biospher packed stainless steel column (250 × 4 mm), and UV-vis detector (Waters 2998). The binary mobile phases were composed of water and acetonitrile with 0.1% acetic acid. In the mobile phase a binary system was applied consisting of A (water) and B (acetonitrile) with the gradient from 85:15 (A:B, vol.%) to 65:35 (A:B, vol.%) in 50 min. The flow rate was 1 mL/min and the injection volume 20 µL. The substances were detected by comparing the spectra and retention times of the samples along with standards (Sigma-Aldrich, Prague, Czech Republic), using a Photodiode Array detector. The content of the identified compounds was calculated from peak areas using external calibration at 254 nm.

### 3.4. UHPLC-MS/MS Analysis

The extraction and analytical method for UHPLC-MS/MS analysis of IVM and its metabolites has been described previously in [11]. In total, 50 mg of each sample (soy roots, leaves, pods, and seeds) was extracted and analyzed.

The LC-MS analysis of isoflavone compounds was performed on an UHPLC (Nexera, Shimadzu, Kyoto, Japan) liquid chromatography system, coupled with QqQ mass spectrometer (LC-MS 8030, Kyoto, Japan, Shimadzu). Chromatographic separation was carried out on a Zorbax RRHD Eclipse plus C18 column (150 × 2.1 mm, particle sizes 1.8 μm, Agilent Technologies, Waldbronn, Germany). The column oven temperature was set at 40 °C and the mobile phase flow rate at 0.4 mL/min. The sample injection volume was 0.1 µL. Analytes were separated using gradient elution consisting of water (A) and acetonitrile (B), both with an additional 0.1% (*v*/*v*) formic acid. The linear gradient was set as follows: 0 min—10% B, 2.20 min—30% B, 4 min—50% B, 6 min—100% B with an isocratic hold for 1.30 min followed with equilibration of the system for 3 min. The mass spectrometer ESI ion source parameters were set as follows: capillary voltage in positive ionization mode 4.5 kV, heat block temperature 400 °C, DL line temperature 250 °C, desolvation gas flow rate 12 L/min and nebulizer gas flow rate 3 L/min. Naringenin was chosen as an internal standard. The mass spectrometer was operated in MRM mode, by monitoring selected ion transitions (Supp. 1). All data were acquired using LabSolution LCMS software ver. 5.93 (Shimadzu, Kyoto, Japan).

### 3.5. Antioxidant Enzymes Activity Assay

The roots and leaves of the plants cultivated in vitro were homogenized in liquid nitrogen, extracted in an extraction buffer (50 mM KH_2_PO_4_; pH 7, 0.1 mM EDTA, 1% PVP K 30, 0.5% Triton-X 100), and centrifuged (14,000 *g*, 10 min, 4 °C), with the supernatant used for the subsequent enzyme assays. Protein content in the supernatant was determined according to [31]. The activities of superoxide dismutase (SOD), catalase (CAT), glutathione S-transferase (GST), ascorbate peroxidase (APX), and guaiacol-specific peroxidase (POX) were measured spectrophotometrically using the common methods [21]. Glutathione reductase (GR) activity was based on changes of GSSG to GSH together with oxidation of NADPH by measuring the absorbance at 340 nm [32].

The specific activities of SOD, CAT, GR, GST, POX, and APX were calculated per protein unit and expressed as percentages of the respective specific activities in the control plants.

### 3.6. Statistical Analysis

To determine changes in the treated plants, the data were processed using STATISTICA.CZ version 12.0 (StatSoft, Prague, Czech Republic) and GraphPad Prism software version 8.4.3 (San Diego, CA, USA). The results are present as the arithmetic mean ± standard deviation (SD). Statistical differences were evaluated by a one-way ANOVA, followed with a post-hoc Tukey’s test. A value of *p* < 0.05 was considered significant.

## 4. Conclusions

Our results show that, for the first time, the anthelmintic drug IVM enters soybean roots and leaves, but not the beans. Therefore, manure, or biosolids containing IVM and its metabolites, does not represent a significant risk of these pharmaceuticals entering the food consumed by humans. However, the presence of these drugs in plants can affect plant yield and secondary metabolism, including the production of polyphenols, pharmaceutically important compounds. In addition, anthelmintically active IVM and its metabolites in roots and leaves of soybean may adversely affect herbivorous invertebrates. The work was performed on a laboratory scale with one type of anthelmintic and only one type of crop. A field study with other anthelmintics and other crops must be performed to publish any recommendations to farmers regarding the handling of manure from treated animals.

## Figures and Tables

**Table 1:** Description of the metabolites of ivermectin (IVM) present in *Glycine max* detected by UHPLC-MS/MS.

t_R_ [min]	*m*/*z* Values of [M + NH_4_]^+^ ions	Molecular Formula	Description of Metabolite Formation	Product ion [M + H]^+^, *m*/*z*	Metabolite Designation
11.774	878	C_47_H_72_O_14_	-CH_2_	307, 551	M1
12.422	908	C_48_H_74_O_15_	+ O	307, 567	M2
12.483	908	C_48_H_74_O_15_	+ O	307, 567	M3
13.096	908	C_48_H_74_O_15_	+ O	307, 567	M4
13.929	892	C_48_H_78_O_14_	-	137, 307, 569	IVM

## Abbreviations

UHPLC–MS/MS	Ultra-high performance liquid chromatography tandem mass spectrometry
SOD	superoxide dismutase
CAT	catalase
GST	glutathione S-transferase
APX	ascorbate peroxidase
POX	guaiacol-specific peroxidase
ESI	electrospray ionization
DMSO	dimethyl sulfoxide
MRM	multiple reaction monitoring
GSH	glutathione
GSSG	glutathione disulfide
NADPH	nicotinamide adenine dinucleotide phosphate
ANOVA	Analysis of variance
